# The Development of Principles for Patient and Public Involvement (PPI) in Preclinical Spinal Cord Research: A Modified Delphi Study

**DOI:** 10.1111/hex.14130

**Published:** 2024-07-04

**Authors:** Pádraig Carroll, Éimear Smith, Adrian Dervan, Ciarán McCarthy, Ian Woods, Cliff Beirne, Geoff Harte, Dónal O'Flynn, John Quinlan, Fergal J. O'Brien, Michelle Flood, Frank Moriarty

**Affiliations:** ^1^ School of Pharmacy and Biomolecular Science RCSI University of Medicine and Health Sciences Dublin Ireland; ^2^ Tissue Engineering Research Group (TERG), Department of Anatomy and Regenerative Medicine RCSI University of Medicine and Health Sciences Dublin Ireland; ^3^ Advanced Materials and BioEngineering Research (AMBER) Centre, Trinity College Dublin (TCD) RCSI University of Medicine and Health Sciences Dublin Ireland; ^4^ National Rehabilitation Hospital Dublin Ireland; ^5^ c/o Irish Rugby Football Union Charitable Trust Dublin Ireland; ^6^ Sports Surgery Clinic Santry Dublin Ireland; ^7^ Tallaght University Hospital, Tallaght Dublin Ireland; ^8^ RCSI PPI Ignite Network Office part of the National PPI Ignite Network based at the University of Galway Galway Ireland

**Keywords:** laboratory research, patient and public engagement, patient and public involvement, preclinical research, spinal cord injury, spinal cord repair

## Abstract

**Introduction:**

There is currently limited guidance for researchers on Patient and Public Involvement (PPI) for preclinical spinal cord research, leading to uncertainty about design and implementation. This study aimed to develop evidence‐informed principles to support preclinical spinal cord researchers to incorporate PPI into their research.

**Methods:**

This study used a modified Delphi method with the aim of establishing consensus on a set of principles for PPI in spinal cord research. Thirty‐eight stakeholders including researchers, clinicians and people living with spinal cord injury took part in the expert panel. Participants were asked to rate their agreement with a series of statements relating to PPI in preclinical spinal cord research over two rounds. As part of Round 2, they were also asked to rate statements as essential or desirable.

**Results:**

Thirty‐eight statements were included in Round 1, after which five statements were amended and two additional statements were added. After Round 2, consensus (> 75% agreement) was reached for a total of 27 principles, with 13 rated as essential and 14 rated as desirable. The principles with highest agreement related to diversity in representation among PPI contributors, clarity of the purpose of PPI and effective communication.

**Conclusion:**

This research developed a previously unavailable set of evidence‐informed principles to inform PPI in preclinical spinal cord research. These principles provide guidance for researchers seeking to conduct PPI in preclinical spinal cord research and may also inform PPI in other preclinical disciplines.

**Patient and Public Involvement Statement:**

This study was conducted as part of a project aiming to develop PPI in preclinical spinal cord injury research associated with an ongoing research collaboration funded by the Irish Rugby Football Union Charitable Trust (IRFU CT) and the Science Foundation Ireland Centre for Advanced Materials and BioEngineering Research (SFI AMBER), with research conducted by the Tissue Engineering Research Group (TERG) at the RCSI University of Medicine and Health Sciences. The project aims to develop an advanced biomaterials platform for spinal cord repair and includes a PPI Advisory Panel comprising researchers, clinicians and seriously injured rugby players to oversee the work of the project. PPI is included in this study through the involvement of members of the PPI Advisory Panel in the conceptualisation of this research, review of findings, identification of key points for discussion and preparation of the study manuscript as co‐authors.

## 1 Introduction

Public Involvement in research is widely defined as research ‘with’ or ‘by’ members of the public, rather than ‘to’, ‘about’ or ‘for’ them’ [1]. Also commonly referred to as Patient and Public Involvement (PPI) [2], it involves a series of approaches that facilitate collaboration or partnership with patients/people with lived experience, carers, service users, families or the public in planning, designing, managing, conducting, dissemination and translation of research [3]. Researchers report incorporating PPI for several reasons including potential benefits to their research such as increasing its relevance [4], ensuring that patients/public can contribute to decisions about the way research is conducted, particularly where public funding is involved [5], and building respect, transparency and trust between researchers and the public [6].

To date, PPI has been most commonly associated with clinical research such as health services research [4, 7, 8] and clinical trials [9, 10]. This is partially due to major health research funders’ increasing requirements to include PPI in grant applications [11, 12, 13]. From a practical perspective, clinical studies involve human participants—often patients living with a particular disease of interest. These individuals can be readily available to research teams and can offer valuable insights into patient priorities and experiences, thus informing the research process.

Comparatively, there has been more limited development of PPI in preclinical research (an umbrella term for basic, fundamental, biomedical, translational or laboratory‐based research) [14, 15]. Several barriers to PPI that are specific to preclinical research have been reported, including communication difficulties between preclinical scientists and PPI contributors [16], that time spent on PPI means time taken away from laboratory work [16, 17] and concerns around the lack of clear links between laboratory‐based research and people's daily lives increasing the risk of tokenism [17, 18, 19]. Another key challenge is the current lack of evidence‐informed guidance on how to implement PPI in preclinical research [14, 16, 20].

Our team (comprising laboratory‐based researchers, physicians, surgeons and seriously injured rugby players living with spinal cord injury [SCI]) initially observed the lack of guidance when aiming to develop a PPI strategy for a preclinical spinal cord repair research project [21, 22]. A scoping review later conducted by the team confirmed that there is limited evidence for preclinical PPI [14]. This was also confirmed by a similar scoping review conducted by researchers in Canada [15]. While some work has been conducted to develop frameworks to support PPI in preclinical research [16, 23, 24], there is still a lack of guidance compared with clinical research that has multiple available PPI frameworks [25]. PPI in preclinical research presents different challenges to clinical research. The results of both scoping reviews show that there is limited literature and no evidence‐informed frameworks for preclinical PPI [14, 15]. Therefore, there was a need to develop an evidence‐informed, consensus‐based framework tailored to support preclinical researchers with PPI. Furthermore, to the best of our knowledge, none have been designed specifically for preclinical SCI research.

Therefore, this study aimed to build on previous research to address this gap and develop a set of evidence‐informed principles for PPI in preclinical spinal cord research. This Delphi study is conducted to support preclinical SCI researchers nationally and internationally to conduct evidence‐informed PPI.

## 2 Methods

This study used a modified Delphi method to achieve its research aim and is reported using the ACCORD (ACcurate COnsensus Reporting Document) Delphi reporting guidelines [26] (Appendix SA). Ethical approval was provided by the Human Research Ethics Committee (REC) at the RCSI University of Medicine and Health Sciences (Record ID: 202211033) based on the study protocol. No protocol was registered in advance of this study. All participants provided written informed consent before participating in the study.

A Delphi method involves assembling an expert panel that completes multiple rounds of a survey, with feedback on group responses between rounds, to achieve consensus on a topic [27]. For this Delphi study, a panel of experts was formed comprising key stakeholders for PPI in preclinical spinal cord research to assist in the development of principles for PPI. The survey was conducted asynchronously and anonymously, to facilitate wide participation of relevant stakeholders and to reduce the potential for power dynamics between stakeholder groups to influence the consensus process.

### 2.1 Participants

A multidisciplinary panel was formed to represent the three groups likely to be involved in PPI in preclinical spinal cord research.

1. People affected by SCI, their families/carers.
2. Preclinical researchers (henceforth referred to as researchers) working in the area of SCI. In this study, ‘preclinical researcher’ refers to researchers who work primarily in non‐patient‐facing laboratory settings. These researchers are different from ‘clinical’ researchers who work with human participants in areas such as health and social care research. Recognising the different degrees of experience, researchers across various career stages were targeted (Principal Investigator, Research Fellow, Postdoctoral Researcher, PhD Student). Researchers had to have 6 months of experience of working in SCI research to be eligible for this study.
3. Medical professionals (physicians/surgeons) involved in treating SCI (henceforth referred to as clinicians). While many healthcare professionals are involved in SCI care, clinicians have a core role and are often involved in research or as members of steering/advisory groups, so they were identified as the key groups for this study.

While there is no agreement on what constitutes the optimum size of a Delphi panel [28], research suggests that 30 participants are sufficient and that recruiting more may not necessarily improve the quality of the Delphi results [29]. We thus aimed to recruit a minimum of 30 experts for the panel, with at least 10 from each identified participant group.

A purposive sampling approach was used to identify and recruit expert panel participants. People affected by SCI were identified via charities supporting seriously injured rugby players. These were the Irish Rugby Football Union (IRFU) Charitable Trust, the (English) Rugby Football Union (RFU) Injured Players Foundation, the (Scottish) Murrayfield Injured Players’ Foundation and the Welsh Rugby Charitable Trust. The charities acted as gatekeepers and agreed to circulate an invitation email among their clients.

Researchers were identified through relevant publications and via professional networks of the research team. Relevant publications were identified by searching Google Scholar for preclinical spinal cord research papers. Potentially eligible researchers were then contacted via their corresponding author email addresses and sent an invitation email. Principal Investigators from research groups conducting preclinical SCI research identified via the professional networks of the research team were also asked to circulate an invitation email amongst their teams. Snowball sampling was also used, where researchers already recruited to the expert panel were asked to share the study invitation with their colleagues. Finally, researchers who had taken part in a previous interview study on PPI conducted by the research team [30] and consented to be re‐contacted about future studies were invited to join the expert panel.

Clinicians were identified via the professional networks of the research team, who work alongside other physicians and surgeons who specialise in SCI. Potentially eligible participants were sent an invitation email by a member of the research team. As for researchers, clinicians who had taken part in a previous interview study and consented to be re‐contacted about future studies were also invited to join the expert panel [30].

The invitation email sent to potential participants, with an attached participant information leaflet, described the study aim, procedures, timelines and expected time commitment, and explained that their involvement and answers would remain confidential. A link to the electronic study consent form was also included, requiring completion before the online Delphi survey could be accessed. Consent from responses was recorded separately from Delphi responses to ensure that participants’ responses remained anonymous. In total, study invitations were circulated to over 150 people affected by SCI, 19 preclinical research groups, eight researchers identified through searching published studies and 25 clinicians. As an incentive for study participation, participants who completed both rounds of the Delphi survey were offered entry into a draw to win one of three €100 gift vouchers.

### 2.2 Statement Generation

The project team had previously conducted a scoping review of the literature on PPI in preclinical research [14] and interview studies with people affected by SCI, researchers and clinicians [30, 31]. The findings of these studies outlined considerations for researchers incorporating PPI into preclinical SCI research and formed the basis of the statements included in the Delphi survey. Additional statements were then added by searching the published literature for principles or guidance related to PPI in existing fields where it is more established, extracting relevant principles not already included and adapting them for preclinical SCI research. Details of the database search for additional guidance are included in Appendix SB.

P.C., a PhD student completing a PhD focused on PPI in preclinical research, generated the statements as described above, with oversight and feedback provided by supervisors MF, who has extensive experience in PPI, and FM, who has extensive experience in the Delphi method. The initial statement list used in Round 1 of the Delphi survey contained 38 statements (Appendix SC). Due to the large number, statements were grouped under overarching domains generated by the research team for the Delphi survey to aid clarity for participants.

### 2.3 Delphi Survey

Participants were first asked to provide demographic information by indicating whether they were a person affected by SCI, a researcher or a clinician and how long they were a member of that participant group. In the Round 1 Delphi survey, participants indicated their agreement with each statement using a 5‐point Likert scale ranging from 1 (*strongly disagree*) to 5 (*strongly agree*), with a midpoint of 3 (*neither agree nor disagree*) facilitating a neutral response. Given the focus of the study, some participants having an ambivalent or uncertain position would be expected and acceptable, and therefore, a midpoint was provided. Each survey page included statements grouped under one domain. Two free‐text boxes were provided at the end of each domain section for participants to explain the rationale for their ratings and suggest changes to existing statements or new statements to include.

For the Round 2 invitation, participants were sent an invitation to complete the Round 2 survey and also a summary of responses from Round 1 as feedback. This summary contained histograms showing the distribution of ratings per statement at Round 1, a synopsis of the percentage agreeing, rationales provided for each statement and any modifications made to statements based on comments from Round 1. Participants were asked to read this Round 1 summary, reflect upon the responses of the wider panel and then complete the updated Round 2 Delphi survey. All statements from Round 1 (with modifications/additions) were included in Round 2. Consensus was defined as > 75% of participants agreeing (selecting 4 [*agree*] or 5 [*strongly agree*]) that a statement is a key principle for PPI in preclinical spinal cord research, and those reaching consensus were included in the final set of key principles. The research team anticipated high numbers of principles reaching consensus. Therefore, at the end of the Round 2 survey, participants were asked to rate each statement (assuming that it reached consensus) as either ‘essential’ (vital for PPI in preclinical spinal cord research) or ‘desirable’ (recommended for PPI in preclinical spinal cord research but not always necessary or possible).

The Round 1 survey was proofed during its development with one researcher external to the study team, selected on the basis of their availability and previous experience with PPI, to ensure that survey content loaded correctly and identify typographical errors. As the sources used to generate statements in this survey included interview studies with all three participant groups providing their perspectives on PPI [30, 31], no specific piloting was conducted to generate new statements.

### 2.4 Data Analysis

Data analysis was conducted using Stata Statistical Software 17 [32]. Percentage agreement with each statement was summarised, as were medians and interquartile ranges of ratings, to describe the distribution amongst the expert panel. Results are presented for ratings per statement at Rounds 1 and 2, change in percentage agreement between rounds and percentage rating as essential. Subgroup analysis is also presented for percentage agreement at each round by participant group.

## 3 Results

### 3.1 Expert Panel

**Figure 1:**
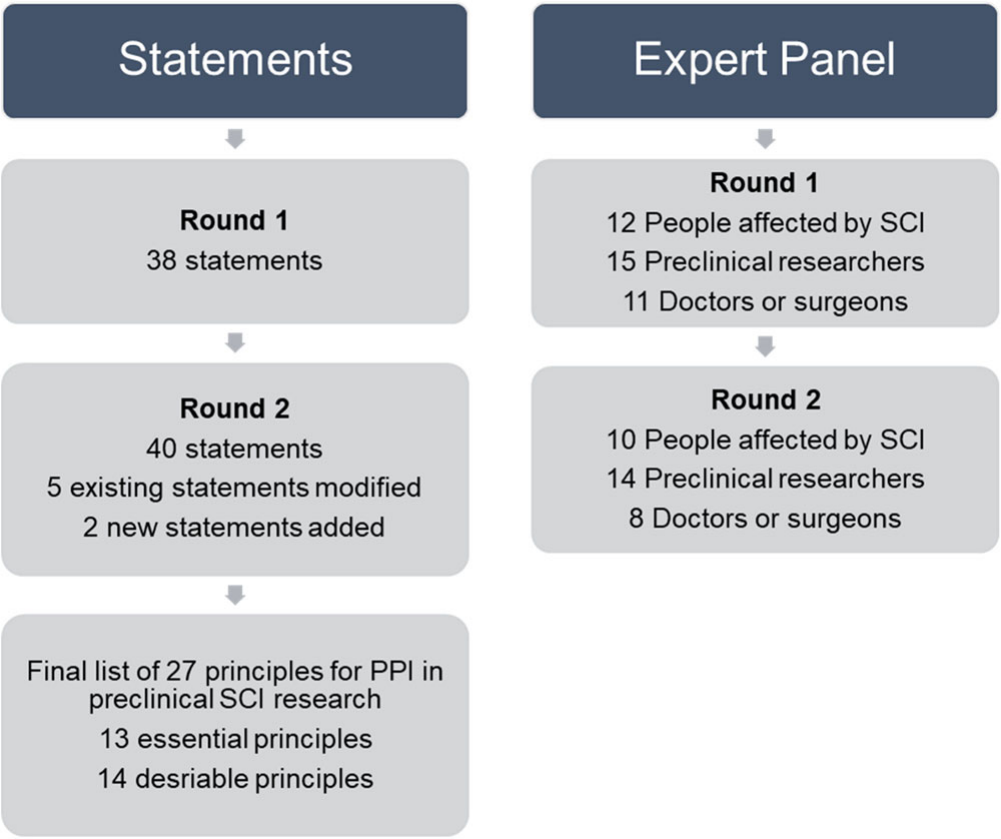
Summary overview of the Delphi procedure.

A total of 38 participants completed Round 1 of the Delphi survey between October and December 2023. Participants included 12 people affected by SCI (median 25.5 years in group; range 15–46 years), 15 preclinical researchers (median 4 years in group; range 1–22 years) and 11 clinicians (median 14 years in group; range 6 months–26 years). All participants from Round 1 were invited to complete Round 2 of the Delphi survey. Round 2 was completed between December 2023 and January 2024 and included 32 participants, retaining 84% of the Round 1 participants, specifically 10 people affected by SCI (median 26 years in group; range 15–46 years), 14 preclinical researchers (median 3.5 years in group; range 1–5 years) and eight clinicians (median 14 years in group; range 4–26 years). Figure 1 summarises the Delphi process.

### 3.2 Round 1 Ratings

In Round 1, participants’ ratings across statements ranged from 5.3% to 100%. Twenty‐three statements had agreement of > 75%. Three statements (Statements 3, 5 and 16) relating to representation among PPI contributors and transparency about project/study progress had agreement of 100%. Statements that had lower agreement (< 50%) included Statement 1: *Recruitment of all those involved in PPI should be finalised before commencing PPI activities* (44.7%), Statement 2: *Researchers should only recruit PPI contributors through spinal cord charities/organisations* (5.3%) and Statement 34: *PPI contributors should be offered financial payment for their time taking part in PPI* (36.8%).

### 3.3 Changes following Round 1

After the Round 1 survey, five existing statements were modified and two new statements were added based on participants’ rationale for their ratings and suggestions for modifications/new statements. Most statements received comments from participants explaining their rationale for their ratings. However, where no clear modification was suggested, the statement was left unchanged for Round 2.

Several participants suggested that Statement 1: *Recruitment of all those involved in PPI should be finalised before commencing PPI activities* should be altered to allow for recruitment to remain ongoing, as its initial form may have been exclusionary. Therefore, Statement 1 was altered to *Recruitment of all those involved in PPI should ideally be finalised before commencing PPI activities; however, this should be balanced against the risk of excluding people who would like to be involved*.

Another suggestion from the panel was to modify Statement 3: *People of different ages should be represented among PPI contributors* to include gender, to ensure a more diverse range of views among PPI contributors. Therefore, Statement 3 was altered to: *A range of demographics, for example, in terms of age and gender, should be represented among PPI contributors*.

Some participants reported that for Statement 8: *PPI contributors should receive PPI training before taking part in PPI*, receiving training may bias PPI contributors when it is their natural opinions that should be valued. Based on participants’ suggestions, Statement 8 was changed to: *PPI contributors should receive PPI training to prepare and support them in contributing their own perspectives to the project or study*.

For Statement 11: *PPI contributors should be offered training and opportunities to conduct preclinical research as part of the research project or study*, participants reported that this may be difficult or impractical for preclinical spinal cord research and may dissuade people from becoming involved in PPI. On this basis, Statement 11 was changed to: *PPI contributors should be offered training and opportunities to carry out preclinical research as part of the project or study where practical and desired by the PPI contributors*.

For Statement 20: *There should be an induction session for all those involved in PPI to meet one another*, participants noted that an induction session could foster a positive atmosphere and help people feel comfortable in PPI and suggested that these sessions should be separate from regular PPI meetings. Based on this suggestion, Statement 20 was changed to: *There should be an induction session for all those involved in PPI to meet one another, before commencing regular PPI meetings*.

Participants also proposed two new statements to be included. One suggestion was to inform people affected by SCI whether there would be any physical demands expected of them when taking part in PPI. On this basis, the following statement was added for Round 2: *New Statement A: The recruitment process for PPI should explain what would be expected of people affected by spinal cord injury who agree to be involved, including any physical demands*. There was also a suggestion to encourage researchers to publish research using routes accessible to more general audiences (e.g., through charities or social media) and to tailor their language and terminology for those audiences. Based on this suggestion, the following statement was added: *New Statement B: Key research findings published in journals should also be disseminated using routes and language suited for non‐scientific audiences*. A summary of Round 1 ratings, participant‐reported rationales for their ratings and details of statement modifications/additions are included in Appendix SD, which was the feedback sent to participants with the Round 2 survey invitation.

### 3.4 Round 2 Ratings

In Round 2, participants’ ratings across statements ranged from 3.1% to 100% agreement. Twenty‐seven statements had agreement of > 75% and one statement (Statement 5: *People with varying severities of spinal cord injury should be represented among PPI contributors*) had agreement of 100%. Statements that had lower agreement (< 50%) included Statement 2: *Researchers should only recruit PPI contributors through spinal cord charities/organisations* (3.1%), Statement 34: *PPI contributors should be offered financial payment for their time taking part in PPI* (37.5%) and Statement 35: *PPI contributors should be offered funding to attend scientific conferences* (43.8%). Based on the Round 2 ratings, the twenty‐seven statements that had agreement > 75% were considered to have reached consensus. Table 1 includes a summary of ratings in Round 1 and Round 2.

**Table 1:** Delphi Round 1 and 2 results: Median, interquartile range, agreement, change between Rounds 1 and 2 and essential rating.

	Round 1 (38 participants)		Round 2 (32 participants)			
**Statement**	**Median (IQR)**	**% Agree**	**Median (IQR)**	**% Agree**	**Change from R1–R2 (%)**	**Rated essential (%)**
*Recruiting PPI contributors*						
1. Recruitment of all those involved in PPI should ideally be finalised before commencing PPI activities; however, this should be balanced against the risk of excluding people who would like to be involved^a^	4 (3, 4)	44.7	4 (3, 4)	71.9	27.2	
2. Researchers should only recruit PPI contributors through spinal cord charities/organisations	2 (2, 4)	5.3	2 (1, 2)	3.1	−2.2	
3. A range of demographics, e.g., in terms of age and gender, should be represented among PPI contributors^a^	4 (3, 5)	100	5 (5, 5)	**96.9**	−3.1	81.3
4. People from different geographical areas should be represented among PPI contributors	5 (4, 5)	92.1	5 (4, 5)	**93.8**	1.7	53.1
5. People with varying severities of spinal cord injury should be represented among PPI contributors	5 (5, 5)	100	5 (4, 5)	**100**	0	81.3
6. People with various lengths of time since having a spinal cord injury should be represented among PPI contributors	5 (4, 5)	97.4	5 (5, 5)	**96.9**	−0.5	78.1
New Statement A. The recruitment process for PPI should explain what would be expected of people affected by spinal cord injury who agree to be involved, including any physical demands^a^			5 (4, 5)	**93.8**	N/A	84.4
*Training*						
7. Researchers should receive PPI training before taking part in PPI	4 (4, 5)	89.5	4 (4, 5)	**90.6**	1.1	53.1
8. PPI contributors should receive PPI training to prepare and support them in contributing their own perspectives to the project or study^a^	4 (3, 5)	63.2	4 (4, 5)	**81.3**	18.1	43.8
9. Any other people involved in PPI should receive PPI training before taking part in PPI	4 (3, 4)	68.4	4 (4, 5)	**81.3**	12.9	25
10. Education on scientific research should be offered to PPI contributors alongside the research project or study	4 (4, 5)	84.2	4 (4, 5)	**90.6**	6.4	25
11. PPI contributors should be offered training and opportunities to carry out preclinical research as part of the project or study where practical and desired by the PPI contributors^a^	4 (3, 4)	52.6	4 (3, 4)	56.3	3.7	
*Agreeing on ways of working together*						
12. Researchers should define and explain PPI to all those involved in PPI	4 (4, 5)	89.5	4 (4, 5)	**96.9**	7.4	84.4
13. All those involved in PPI should agree on how input from PPI contributors will be used to impact the research project or study	4 (4, 5)	86.8	4 (4, 5)	**87.5**	0.7	40.6
14. There should be agreement from all those involved in PPI on their roles in the research project or study	4 (4, 5)	89.5	4 (4, 5)	**90.6**	1.1	62.5
15. PPI contributors should be allowed to provide input using a format of their choosing, e.g., written or verbal, virtual or in person	4 (4, 5)	84.2	4 (4, 5)	**90.6**	6.4	50
*Communicating with one another*						
16. Researchers should be transparent with all those involved in PPI about the progress of the scientific research project or study	5 (4, 5)	100	5 (4, 5)	**93.8**	−6.2	78.1
17. Researchers should explain the timelines for translating preclinical research into clinical practice	4.5 (4, 5)	89.5	4.5 (4, 5)	**90.6**	1.1	53.1
18. All those involved in PPI should be able to provide feedback on the research project or study progress to researchers	4 (4, 5)	92.1	4 (4, 5)	**90.6**	−1.5	50
19. PPI contributors should be informed whether there would be potential benefits from their involvement in the research project or study or the outputs that it produces	5 (4, 5)	97.4	4 (4, 5)	**90.6**	−6.8	59.4
*Conducting PPI activities*						
20. There should be an induction session for all those involved in PPI to meet one another, before commencing regular PPI meetings^a^	4 (3, 4.25)	60.5	4 (3, 5)	68.8	8.3	
21. PPI activities should create opportunities for all those involved in PPI to interact with one another	4 (3, 4.25)	65.8	4 (3, 5)	68.8	3	
22. PPI activities should include identifying common areas of research interest between all those involved in PPI	4 (3, 4)	71	4 (3, 5)	68.8	−2.2	
23. PPI activities should include identifying goals for preclinical/laboratory‐based spinal cord research	4 (4, 5)	79	4 (4, 5)	**81.3**	2.3	43.8
24. PPI activities should include opportunities for PPI contributors to share experiences of their condition	4 (3.75, 5)	76.3	4 (4, 5)	**81.3**	5	43.8
25. There should be at least two PPI meetings held every year for the duration of the research project or study	4 (3, 4.25)	65.8	4 (3, 4)	62.5	−3.3	
26. PPI in preclinical spinal cord research should take place using virtual/hybrid platforms (e.g., Zoom/Teams)	3.5 (3, 4)	50	4 (3, 4)	59.4	9.4	
*Evaluation and dissemination*						
27. Researchers should describe PPI within the research project or study in their research publications	4 (4, 4.25)	81.6	4 (4, 4.75)	**84.4**	2.8	40.6
28. Researchers should present key findings in their research publications using language suited for non‐scientific audiences	4 (3, 5)	60.5	4 (3.25, 5)	75	14.5	
New Statement B: Key research findings published in journals should also be disseminated using routes and language suited for non‐scientific audiences^a^			4.5 (4, 5)	**90.6**	N/A	62.5
29. Researchers should evaluate PPI throughout the research project or study	4 (3.75, 4)	76.3	4 (4, 4.75)	**93.8**	17.5	40.6
30. Researchers should disseminate the impact of PPI upon the research project or study	4 (4, 5)	84.2	4 (4, 5)	**81.3**	−2.9	31.3
31. PPI should involve developing information resources on preclinical research using language suited for non‐scientific audiences	4 (4, 5)	89.5	4 (4, 5)	**96.9**	7.4	40.6
*Supporting PPI contributors*						
32. One member of the research team should be responsible for PPI within the research project or study	4 (3, 4)	55.3	3.5 (3, 4)	50	−5.3	
33. Researchers should ensure that funding is in place to support all aspects of PPI planned within the research project or study	4 (3.75, 4)	76.3	4 (4, 5)	**84.4**	8.1	46.9
34. PPI contributors should be offered financial payment for their time taking part in PPI	3 (3, 4)	36.8	3 (2.25, 4)	37.5	0.7	
35. PPI contributors should be offered funding to attend scientific conferences	4 (3, 4.25)	55.3	3 (3, 4)	43.8	−11.5	
36. Researchers should prioritise ease of access to in‐person PPI activities	4 (4, 5)	78.9	4.5 (4, 5)	**90.6**	11.7	62.5
37. PPI contributors should be offered transport to/from in‐person PPI activities	4 (3.75, 5)	76.3	4 (4, 5)	**81.3**	5	40.6
38. PPI contributors should be offered emotional support when taking part in PPI	4 (3, 4)	57.9	4 (3, 5)	59.4	1.5	

*Note:* Bold value indicate reached consensus for inclusion after Round 2.

^a^ Statement was modified/added between Round 1 and Round 2. Modified wording is included in the table; see Appendix SC for original wording of statements at Round 1.

The majority of statements had increased levels of agreement at Round 2, with the largest increase seen in the modified Statement 1: *Recruitment of all those involved in PPI should ideally be finalised before commencing PPI activities; however, this should be balanced against the risk of excluding people who would like to be involved* (+27.1%). However, this increase was still insufficient to reach the threshold for defining consensus. Some statements received lower levels of agreement in Round 2 compared to Round 1, with the largest decrease seen in Statement 35. *PPI contributors should be offered funding to attend scientific conferences* (11.5%).

Other statements that saw high change in agreement (±10%) between Rounds 1 and 2 included the modified Statement 8: *PPI contributors should receive PPI training to prepare and support them in contributing their own perspectives to the project or study* (+18.1%), Statement 9: *Any other people involved in PPI should receive PPI training before taking part in PPI* (+12.9%), Statement 28: *Researchers should present key findings in their research publications using language suited for non‐scientific audiences* (+14.5%), Statement 29: *Researchers should evaluate PPI throughout the research project or study* (+17.5%) and Statement 36: *Researchers should evaluate PPI throughout the research project or study* (+11.7%). Most statements modified between Round 1 and Round 2 had higher levels of agreement after Round 2. However, Statement 3: *A range of demographics, for example, in terms of age and gender, should be represented among PPI contributors*, saw a small decrease in agreement (down 3.1%, to 96.9% agreement), though agreement remained very high. Both new statements added after Round 1 reached the threshold for consensus. Statement 28: *Researchers should present key findings in their research publications using language suited for non‐scientific audiences*, was rated agree/strongly agree by 75% of the expert panel, which was close to the threshold to define consensus, but insufficient.

### 3.5 Agreement by Participant Group

Level of agreement was generally consistent when comparing ratings by participant group (people affected by SCI, researchers or clinicians), though there were some differences in agreement in several of the statements. In Statement 8: *PPI contributors should receive PPI training to prepare and support them in contributing their own perspectives to the project or study*, people affected by SCI had a much lower Round 1 agreement level (33.3%) than researchers (73.3%) and clinicians (81.8%). In Round 2, their level of agreement increased considerably to 70% after the statement was modified between rounds.

In Statement 15: *PPI contributors should be allowed to provide input using a format of their choosing, for example, written or verbal, virtual or in person*, among people affected by SCI, there was an increase in agreement from 66.7% to 90% between rounds. This is distinct from researchers and clinicians, who had high levels of agreement with this statement in both rounds.

There was a difference in agreement levels between participant groups in Statement 20: *There should be an induction session for all those involved in PPI to meet one another, before commencing regular PPI meetings*. Researchers reported high levels of agreement in both Round 1 (93.3%) and Round 2 (85.7%). This contrasts with lower agreement from people affected by SCI (50% in Round 1 and 60% in Round 2). There was much lower agreement with this statement from clinicians who had agreement levels of 27.3% in Round 1% and 50% in Round 2.

A similar comparison can be seen in the following Statement 21: *PPI activities should create opportunities for all those involved in PPI to interact with one another*. Researchers indicated a high level of agreement with this statement in Round 2 (100%). This is considerably different from the levels of agreement among people affected by SCI (40%) and clinicians (50%).

Statement 23: *PPI activities should include identifying goals for preclinical laboratory‐based spinal cord research*, had high levels of agreement for people affected by SCI (80%) and researchers (92.9%). However, clinicians reported lower levels of agreement (62.5%). This disparity was maintained across both rounds.

Statement 24: *PPI activities should include opportunities for PPI contributors to share experiences of their condition*, had varying levels of Round 1 agreement between researchers (93.3%), clinicians (72.7%) and people affected by SCI (58.3%). In Round 2, researchers maintained a high level of agreement (100%) and people affected by SCI had considerably higher agreement than in Round 1 (80%). However, clinicians' agreement levels decreased in Round 2 (50%).

There were further differences seen in Statement 28: *Researchers should present key findings in their research publications using language suited for non‐scientific audiences*. After Round 2, clinicians had lower agreement (50%) than people affected by SCI (80%) or researchers (85.7%). A similar gap was initially seen in Statement 31: *PPI should involve developing information resources on preclinical research using language suited for non‐scientific audiences*. In Round 1, clinicians had lower agreement (63.6%) than researchers (100%) or people affected by SCI (100%). However, there was a considerable increase in agreement in Round 2 (87.5%), with clinicians reaching the threshold for consensus on this statement. Statement 34: *PPI contributors should be offered financial payment for their time taking part in PPI*, had very low levels of agreement at Round 1 among people affected by SCI (16.7%) and clinicians (27.3%). This contrasts with researchers, who had 60% agreement in Round 1. In Round 2, the ratings of people affected by SCI (40%) and researchers (50%) moved closer, and clinicians’ agreement decreased (12.5%).

Statement 35: *PPI contributors should be offered funding to attend scientific conferences*, had agreement of 80% among researchers in Round 1. This contrasts with lower agreement in Round 1 among people affected by SCI (33.3%) and clinicians (45.5%). In Round 2, researchers’ ratings dropped considerably to 42.9%, which was more in line with the Round 2 scores of people affected by SCI (50%) and clinicians (37.5%). Table 2 summarises Rounds 1 and 2 agreements by participant group.

**Table 2:** Percentage agreement of Rounds 1 and 2 by participant group.

	Round 1			Round 2		
	People affected by SCI	Researchers	Clinicians	People affected by SCI	Researchers	Clinicians
*Recruiting PPI contributors*						
1. Recruitment of all those involved in PPI should ideally be finalised before commencing PPI activities; however, this should be balanced against the risk of excluding people who would like to be involved^a^	50	33.3	54.5	**80**	71.4	62.5
2. Researchers should only recruit PPI contributors through spinal cord charities/organisations	16.7	0	0	10	0	0
3. A range of demographics, e.g., in terms of age and gender, should be represented among PPI contributors^a^	100	100	100	**100**	**92.9**	**100**
4. People from different geographical areas should be represented among PPI contributors	91.7	86.7	100	**90**	**92.7**	**100**
5. People with varying severities of spinal cord injury should be represented among PPI contributors	100	100	100	**100**	**100**	**100**
6. People with various lengths of time since having a spinal cord injury should be represented among PPI contributors	100	93.3	100	**100**	**100**	**87.5**
New Statement A. The recruitment process for PPI should explain what would be expected of people affected by spinal cord injury who agree to be involved, including any physical demands^a^	NA	NA	NA	**100**	**92.9**	**87.5**
*Training*						
7. Researchers should receive PPI training before taking part in PPI	83.3	93.3	90.9	70	**100**	**100**
8. PPI contributors should receive PPI training to prepare and support them in contributing their own perspectives to the project or study^a^	33.3	73.3	81.8	70	**85.7**	**87.5**
9. Any other people involved in PPI should receive PPI training before taking part in PPI	50	73.3	81.8	**80**	**78.6**	**87.5**
10. Education on scientific research should be offered to PPI contributors alongside the research project or study	66.7	100	81.8	**80**	**100**	**87.5**
11. PPI contributors should be offered training and opportunities to carry out preclinical research as part of the project or study where practical and desired by the PPI contributors^a^	66.7	53.3	36.4	60	64.3	37.5
*Agreeing on ways of working together*						
12. Researchers should define and explain PPI to all those involved in PPI	91.7	80	100	**100**	**92.7**	**100**
13. All those involved in PPI should agree on how input from PPI contributors will be used to impact the research project or study	83.3	86.7	90.9	**80**	**92.9**	**87.5**
14. There should be agreement from all those involved in PPI on their roles in the research project or study	83.3	93.3	90.9	**80**	**92.9**	**100**
15. PPI contributors should be allowed to provide input using a format of their choosing, e.g., written or verbal, virtual or in person	66.7	93.3	90.9	**90**	**85.7**	**100**
*Communicating with one another*						
16. Researchers should be transparent with all those involved in PPI about the progress of the scientific research project or study	100	100	100	**100**	**92.9**	**87.5**
17. Researchers should explain the timelines for translating preclinical research into clinical practice	75	93.3	100	**100**	**92.9**	75
18. All those involved in PPI should be able to provide feedback on the research project or study progress to researchers	91.7	86.7	100	**100**	**85.7**	**87.5**
19. PPI contributors should be informed whether there would be potential benefits from their involvement in the research project or study or the outputs that it produces	100	93.3	100	**90**	**92.9**	**87.5**
*Conducting PPI activities*						
20. There should be an induction session for all those involved in PPI to meet one another, before commencing regular PPI meetings^a^	50	93.3	27.3	60	**85.7**	50
21. PPI activities should create opportunities for all those involved in PPI to interact with one another	50	86.7	54.6	40	**100**	50
22. PPI activities should include identifying common areas of research interest between all those involved in PPI	75	80	54.6	60	**85.7**	50
23. PPI activities should include identifying goals for preclinical/laboratory‐based spinal cord research	91.7	86.7	54.5	**80**	**92.9**	62.5
24. PPI activities should include opportunities for PPI contributors to share experiences of their condition	58.3	93.3	72.7	**80**	**100**	50
25. There should be at least two PPI meetings held every year for the duration of the research project or study	58.3	73.3	63.6	50	**78.6**	50
26. PPI in preclinical spinal cord research should take place using virtual/hybrid platforms (e.g., Zoom/Teams)	66.7	40	45.5	50	71.4	50
*Evaluation and dissemination*						
27. Researchers should describe PPI within the research project or study in their research publications	75	80	90.9	70	**85.7**	**100**
28. Researchers should present key findings in their research publications using language suited for non‐scientific audiences	75	66.6	36.4	**80**	**85.7**	50
New Statement B: Key research findings published in journals should also be disseminated using routes and language suited for non‐scientific audiences^a^		NA	NA	**90**	**100**	75
29. Researchers should evaluate PPI throughout the research project or study	83.3	73.3	72.7	**100**	**92.9**	**87.5**
30. Researchers should disseminate the impact of PPI upon the research project or study	83.3	93.3	72.7	70	**100**	62.5
31. PPI should involve developing information resources on preclinical research using language suited for non‐scientific audiences	100	100	63.6	**100**	**100**	**87.5**
*Supporting PPI contributors*						
32. One member of the research team should be responsible for PPI within the research project or study	58.3	60	45.6	30	57.1	62.5
33. Researchers should ensure that funding is in place to support all aspects of PPI planned within the research project or study	58.3	86.7	81.8	70	**92.9**	**87.5**
34. PPI contributors should be offered financial payment for their time taking part in PPI	16.7	60	27.3	40	50	12.5
35. PPI contributors should be offered funding to attend scientific conferences	33.3	80	45.5	50	42.9	37.5
36. Researchers should prioritise ease of access to in‐person PPI activities	66.7	86.67	81.8	**80**	**100**	**87.5**
37. PPI contributors should be offered transport to/from in‐person PPI activities	66.7	73.3	90.9	**80**	**92.9**	62.5
38. PPI contributors should be offered emotional support when taking part in PPI	58.3	60	54.5	50	71.4	50

*Note:* Bold values indicate participant group agreement at Round 2 above the threshold used for defining consensus.

^a^ Statement was modified/added between Round 1 and Round 2. Modified wording is included in the table; see Appendix SC for original wording of statements at Round 1.

### 3.6 Essential Versus Desirable Rating

Table 1 shows the percentage of panel members rating each statement as essential. Essential or desirable ratings were only applied to statements that had reached the threshold for inclusion after Round 2. Thirteen statements were considered as essential and fourteen statements were considered as desirable. The final list of essential and desirable principles is displayed in Table 3. The statements with the highest essential rating were E5: *The recruitment process for PPI should explain what would be expected of people affected by spinal cord injury who agree to be involved, including any physical demands*, and E7: *Researchers should define and explain PPI to all those involved in PPI*. Both principles were considered as essential by 84.4% of the panel members. The statements receiving the highest ratings as most votes for desirable were D2: *Any other people involved in PPI should receive PPI training before taking part in PPI*, and D3: *Education on scientific research should be offered to PPI contributors alongside the research project or study*. Both of these statements were rated desirable by 75% of the panel members. Two statements were rated as essential and desirable by exactly 50% of the panel D5: *PPI contributors should be allowed to provide input using a format of their choosing, for example, written or verbal, virtual or in person*, and D6: *All those involved in PPI should be able to provide feedback on the research project or study progress to researchers*. However, as a majority rating a statement as essential was required, these were classified as desirable.

**Table 3:** Essential and desirable principles for PPI in preclinical spinal cord research.

*Essential*	
E1	A range of demographics, e.g., in terms of age and gender, should be represented among PPI contributors
E2	People from different geographical areas should be represented among PPI contributors
E3	People with varying severities of spinal cord injury should be represented among PPI contributors
E4	People with various lengths of time since having a spinal cord injury should be represented among PPI contributors
E5	The recruitment process for PPI should explain what would be expected of people affected by spinal cord injury who agree to be involved, including any physical demands
E6	Researchers should receive PPI training before taking part in PPI
E7	Researchers should define and explain PPI to all those involved in PPI
E8	There should be agreement from all those involved in PPI on their roles in the research project or study
E9	Researchers should be transparent with all those involved in PPI about the progress of the scientific research project or study
E10	Researchers should explain the timelines for translating preclinical research into clinical practice
E11	Key research findings published in journals should also be disseminated using routes and language suited for non‐scientific audiences
E12	PPI contributors should be informed whether there would be potential benefits from their involvement in the research project or study or the outputs that it produces
E13	Researchers should prioritise ease of access to in‐person PPI activities
*Desirable*	
D1	PPI contributors should receive PPI training to prepare and support them in contributing their own perspectives to the project or study
D2	Any other people involved in PPI should receive PPI training before taking part in PPI
D3	Education on scientific research should be offered to PPI contributors alongside the research project or study
D4	All those involved in PPI should agree on how input from PPI contributors will be used to impact the research project or study
D5	PPI contributors should be allowed to provide input using a format of their choosing, e.g., written or verbal, virtual or in person
D6	All those involved in PPI should be able to provide feedback on the research project or study progress to researchers
D7	PPI activities should include identifying goals for preclinical/laboratory‐based spinal cord research
D8	PPI activities should include opportunities for PPI contributors to share experiences of their condition
D9	Researchers should describe PPI within the research project or study in their research publications
D10	Researchers should evaluate PPI throughout the research project or study
D11	Researchers should disseminate the impact of PPI upon the research project or study
D12	PPI should involve developing information resources on preclinical research using language suited for non‐scientific audiences
D13	Researchers should ensure that funding is in place to support all aspects of PPI planned within the research project or study
D14	PPI contributors should be offered transport to/from in‐person PPI activities

## 4 Discussion

This study aimed to develop key principles for PPI in preclinical spinal cord research to support researchers in conducting evidence‐informed PPI. Using a modified Delphi method, consensus was reached among an expert panel on 27 principles. Thirteen of these principles were considered essential for PPI in preclinical spinal cord research and 14 principles were considered desirable by the expert panel.

Out of 40 statements in total that were considered as part of this study, 27 reached consensus for inclusion after two rounds. There was no consensus on the remaining 13 statements. One statement that did not reach consensus related to offering PPI contributors payment for their time taking part in PPI. This finding was unexpected as for PPI in health research, offering payment is normally considered essential to recognise the contributions of people taking part in PPI and to support people from diverse backgrounds to be involved in research through PPI [33, 34]. While guidelines help, in practice, paying PPI contributors needs to be agreed on an individual basis as some people may not necessarily want to be paid for their time, and for other PPI contributors, accepting payment for PPI may impact their eligibility to receive state entitlements [35]. Recognising the importance and complexity of compensating PPI contributors, specific guidelines have been published for paying people living with SCI for their involvement in research [36]. However, these should be considered in association with national/local guidelines and PPI contributors' preferences.

Statement 35: *PPI contributors should be offered funding to attend scientific conferences*, did not reach consensus and saw a large decrease in agreement between rounds, particularly among researchers. This may have been a result of the Delphi process, whereby seeing and reflecting on the Round 1 summary, researchers in Round 2 reassessed their ratings, leading to lower agreement. According to some PPI guidance, funding PPI contributors to attend conferences can be an alternative to payment, if PPI contributors do not wish to accept payment for their time [37].

Several of the principles that reached consensus for inclusion relate to clearly explaining the role and remit of PPI for preclinical SCI research. Preclinical research generally has long timelines before any potential clinical benefit for people. Therefore, it is important for researchers to be clear and realistic when discussing project progress with PPI contributors to avoid creating unrealistic expectations and the potential for misunderstanding/disappointment [30]. However, this should not necessarily dissuade researchers or PPI contributors from becoming involved in PPI, as previous research has demonstrated that PPI is both possible and desired in preclinical research, and can benefit all those involved [18]. Furthermore, PPI may help address the challenge of preclinical SCI research failing to translate by helping focus research priorities on areas of most clinical relevance to people affected by SCI [38].

An unexpected finding of this study was that researchers had higher levels of agreement than other participant groups on the statements relating to communicating and interacting with PPI contributors. This is encouraging as previous research has suggested that communication is a key factor in preclinical researcher hesitancy to adopt PPI [16]. The findings of our study suggest that researchers may feel that communication with PPI contributors is an important aspect of PPI. This is supported by our previous interview study with researchers, who reported that good communication and rapport are required to establish good relationships with PPI contributors [30]. It may be that researchers hold communication as an important aspect of PPI, without necessarily possessing the training required to work with PPI contributors. Preclinical researchers work primarily in non‐patient‐facing settings and may not necessarily be trained or experienced in communicating directly with non‐researcher stakeholders. Providing PPI training for researchers to communicate and interact with members of the public may help encourage researchers to incorporate PPI into their research. Other studies have suggested that researchers’ communication skills are improved by the process of PPI, helping build rapport, transparency and trust with PPI contributors [6, 18, 39]. It is possible that as researchers engage in PPI, it becomes progressively easier with practice. Therefore, offering initial training could prove beneficial in overcoming potential communication difficulties.

Another difference between participant groups was whether PPI contributors should only be recruited from SCI charities and organisations. No researchers or clinicians agreed with this statement in both rounds of the Delphi survey. However, a small number of people affected by SCI agreed with this statement in both rounds. This finding may have resulted from our sampling strategy, as all participants in the people affected by SCI group were recruited through charities that support seriously injured rugby players. In our previous interview study, individuals with SCI emphasised that their understanding of SCI stemmed from their own first‐hand experiences with the condition [31]. Hence, their viewpoint on recruitment holds significance. The SCI community in Ireland has been described as highly networked [30]. Therefore, this study's findings suggest that further research is needed to explore the recruitment methods for involving PPI contributors in SCI research.

As a large number of principles reached consensus for inclusion, separating them into an essential and desirable list may be useful to identify those most vital principles for PPI in preclinical SCI research. However, principles that were considered desirable still achieved consensus and are therefore important for PPI in preclinical research. Many of the desirable principles are fundamental aspects of PPI such as PPI training or collaborating with PPI contributors on research dissemination [14, 25]. Several of the principles that were considered essential by the panel relate to recruiting a diverse set of PPI contributors. Ensuring that PPI contributors represent a wide range of views is important for PPI in preclinical research, as including only a limited number of perspectives can be harmful by potentially excluding groups of people with highly relevant perspectives [40].

The findings of this study are comparable with other Delphi studies on PPI in clinical research that identified similar principles to those developed in the current study, for example, communicate and inform regularly, accommodate individual and collective needs and evaluate throughout [41]. While there are several similar principles in this Delphi, when compared with those for clinical research, in practical terms, PPI presents different challenges for preclinical researchers, who shoulder most responsibility for PPI according to the principles. It is important to consider these challenges to facilitate implementation. Principles E1–E4 relate to identifying diverse PPI contributors, and yet, we know that preclinical researchers do not routinely have ready access to any patient/public, let alone the diverse mix recommended in these principles. To achieve this, researchers should be encouraged to seek appropriate supports at an institutional level, where available, and make use of regional/national resources, for example, People in Research in the United Kingdom [42] or the PPI Ignite Network PPI Opportunities Noticeboard in Ireland [43], as well as charities/patient organisations and social media. Additionally, many preclinical researchers are not familiar with PPI or are including PPI in their work for the first time [14, 16, 17, 30], and have reported feeling unequipped to communicate and interact with the public in a professional capacity [16]. Given the centrality of the researcher communicating with the public/patients in PPI, including the ability to explain PPI and roles within projects (E7, E8), this makes undertaking training (E6) particularly important for preclinical researchers. Training for preclinical PPI was considered essential for researchers and desirable for PPI contributors. Therefore, capacity‐building activities are important and training opportunities may be available at the institutional, regional or national level. In Ireland, co‐designed templates for both researchers and the public have been developed and made freely available to support the expansion of training opportunities [44, 45]. Researchers may also wish to make use of specific resources developed to support preclinical PPI [24, 46]. Other important sources of support include dedicated PPI staff at some universities as well as institutional champions or experienced colleagues.

One barrier noted specifically for PPI in preclinical SCI research has been the more limited mobility experienced by many people living with SCI [30]. This must be addressed proactively with relevant accommodations to minimise barriers to people living with SCI from becoming involved [31]. Previous guidance has suggested specific strategies that may support researchers to overcome the barrier of limited mobility. While they focus on research participation or supporting people living with SCI to participate in the community, the guidance is helpful for those planning PPI. Examples of these strategies include ensuring that in‐person meetings are held in locations easily navigable for wheelchairs [47], using online meeting formats (e.g., Zoom, Skype, Microsoft Teams) to host meetings so that PPI contributors are not required to travel [48], arranging private travel to in‐person PPI activities as public transportation may not always be well adapted for people with limited mobility [49] and engaging with PPI contributors in a positive and supportive manner to encourage their participation in PPI [50]. Another consideration is to plan PPI meetings/activities around the delivery of care for people affected by SCI living in the community. This may be particularly relevant for those receiving care from carers or nurses visiting their homes [51], as this care may occur early in the day and preclude participation in morning PPI activities.

While existing guidelines are helpful, it is also important for researchers to involve PPI contributors in planning, asking them about their specific needs and tailoring venue choice and PPI activities accordingly. Researchers should be aware that a person living with paraplegia (paralysis affecting the lower body) will have different requirements to a person living with tetraplegia (paralysis from the neck down). This can be addressed by asking potential PPI contributors to advise on the best ways to support their participation in preclinical PPI [52] and by linking in with patient organisations who have experience of working with people affected by SCI.

## 5 Strengths and Limitations

This study had several strengths. The Delphi expert panel comprised key stakeholder groups for PPI in preclinical SCI research. People affected by SCI, preclinical researchers and clinicians possess specific viewpoints and perspectives, meaning that a wide range of views were included. There was a high retention rate for panel members between rounds, strengthening the validity of the findings. Finally, the essential versus desirable rating conducted in this study helps further categorise the large number of principles that reached consensus [53].

This study had several limitations. The key principles developed in this study were focused on PPI in preclinical SCI research, with a focus on rugby‐related SCI in line with the broader study. While many of the principles are applicable to wider preclinical research, they may not all be generalisable to all other areas. The researcher participant group reported the lowest median experience in their group, indicating high participation from early career researchers whose perspectives may differ from experienced principal investigators. Due to time constraints, only two Delphi rounds were conducted. While there is no set number of rounds for a Delphi, holding further rounds may have resulted in some borderline principles reaching consensus [27]. In the essential versus desirable rating, participants were asked to rate each item on the basis that it was included as a key principle. In this section of the Round 2 survey, there was no option for panel members to indicate if they had disagreed with a statement and felt that it was neither essential nor desirable, meaning that participants may have had to rate principles that they disagreed with. In contrast to the neutral option in the agreement ratings, this was not appropriate here, as essential or desirable ratings were considered mutually exclusive and collectively exhaustive options as applied to statements that reached consensus from the whole panel. By conducting the Delphi survey online, digital inclusion is a potential limitation of this study. An online survey had practical advantages including efficiency, feasibility, the ready ability to include international participants and synchronous and anonymous participation, reducing potential for power dynamics. However, there is a possibility that potential participants may have been unable to engage with the digital approach. Finally, no formal piloting was carried out in the development of the Delphi survey.

## 6 Conclusion

This research develops and establishes key principles for PPI in preclinical spinal cord research through the consensus of people affected by SCI, preclinical researchers and clinicians. This study contributes new knowledge by providing evidence‐informed guidelines for preclinical researchers to conduct PPI. The study findings indicate that involving a diverse group of people living with SCI in terms of demographics, injury and geographical location is important and clear communication policies are essential for conducting PPI in SCI research. The participants reached a consensus that PPI training is essential for researchers and that incorporating education into the PPI process is desirable—indicative of the potential for knowledge sharing to overcome existing challenges in PPI.

## Author Contributions

**Pádraig Carroll:** conceptualisation, data curation, formal analysis, investigation, methodology, project administration, visualisation, writing–original draft. **Éimear Smith:** conceptualisation, funding acquisition, methodology, resources, supervision, writing–review and editing. **Adrian Dervan:** writing–review and editing. **Ciarán McCarthy:** conceptualisation, writing–review and editing. **Ian Woods:** writing–review and editing. **Cliff Beirne:** writing–review and editing. **Geoff Harte:** writing–review and editing. **Dónal O'Flynn:** writing–review and editing. **John Quinlan:** writing–review and editing. **Fergal J. O'Brien:** conceptualisation, funding acquisition, methodology, resources, supervision, writing–review and editing. **Michelle Flood:** conceptualisation, formal analysis, funding acquisition, investigation, methodology, supervision, writing–original draft, writing–review and editing. **Frank Moriarty:** conceptualisation, data curation, formal analysis, funding acquisition, investigation, methodology, supervision, writing–original draft, writing–review and editing.

## Ethics Statement

The Human Research Ethics Committee (REC) at the RCSI University of Medicine and Health Sciences provided ethical approval for the study (REC Record ID: 202211033). All participants provided written informed consent before participating in the study.

## Consent

No copyrighted material has been reproduced from other sources and no permission was required. The sources cited in this study are referenced in the study manuscript.

## Conflicts of Interest

The authors declare no conflicts of interest.

## Supporting information

Supporting information.

Supporting information.

Supporting information.

Supporting information.

## Data Availability

All data generated or analysed during the study are included in this published article and its supplementary information files.
