# Supplementary material for: The Development of Principles for Patient and Public Involvement (PPI) in Preclinical Spinal Cord Research: A Modified Delphi Study
Source: Health Expect. 2024 Jul 4;27(4):e14130. doi: 10.1111/hex.14130 (PMC11222973; doi:10.1111/hex.14130)
Supplement: Supplementary file 2 — Supporting information. [file HEX-27-e14130-s004.docx]

**Appendix B:** Search terms and screening process for papers containing additional PPI guidance

| **EMBASE/CINAHL/Medline/APA** | No. of studies |
| --- | --- |
| **Concept 1: Patient and Public Involvement**  (patient and public involvement or patient and public engagement)  **Concept 2: Principles or Guidelines**  (guidelines or best practices or protocols or principles) |  |
| **Search terms:** (patient and public involvement or patient and public engagement) AND (guidelines or best practices or protocols or principles) | 1123 |
| Duplicates removed | 226 |
| Papers assessed for eligibility by title and abstract | 897 |
| Papers excluded | 861 |
| Studies included from database search | 36 |
| Further studies identified | 4 |
| **Final number of studies included** | **40** |
