# Supplementary material for: The Development of Principles for Patient and Public Involvement (PPI) in Preclinical Spinal Cord Research: A Modified Delphi Study
Source: Health Expect. 2024 Jul 4;27(4):e14130. doi: 10.1111/hex.14130 (PMC11222973; doi:10.1111/hex.14130)
Supplement: Supplementary file 3 — Supporting information. [file HEX-27-e14130-s001.docx]

**Appendix C:** Round 1 Delphi Statements

| **No.** | **Statements** |
| --- | --- |
|  | **Recruiting PPI Contributors** |
| **1** | Recruitment of anyone involved in PPI should be finalised before commencing PPI activities |
| **2** | Researchers should only recruit PPI contributors through spinal cord charities/ organisations |
| **3** | People of different ages should be represented among PPI contributors |
| **4** | People from different geographical areas should be represented among PPI contributors |
| **5** | People with varying severities of spinal cord injury should be represented among PPI contributors |
| **6** | People with varying time durations since obtaining a spinal cord injury should be represented among PPI contributors |
|  | **Training** |
| **7** | Researchers should receive PPI training before taking part in PPI |
| **8** | PPI contributors should receive PPI training before taking part in PPI |
| **9** | Clinicians should receive PPI training before taking part in PPI |
| **10** | Education on scientific research should be offered to PPI contributors alongside the research project or study (i.e. visit research labs/ attend seminars on current research) |
| **11** | PPI contributors should be offered training and opportunities to conduct preclinical research as part of the research project or study |
|  | **Agreeing on Ways of Working Together** |
| **12** | Researchers should define and explain PPI to everyone involved |
| **13** | Everyone involved should agree on how input from PPI contributors will be used to impact the research project or study |
| **14** | There should be agreement from everyone involved on their role in PPI |
| **15** | PPI contributors should be allowed to provide input using a format of their choosing i.e. written or verbal, virtual or in-person |
|  | **Communicating with One Another** |
| **16** | Researchers should be transparent to all involved about the progress of the scientific research project or study |
| **17** | Researchers should explain the timelines for translating preclinical research into clinical practice |
| **18** | Everyone involved in PPI should be able to provide feedback on the project or study progress to researchers |
| **19** | Researchers should inform PPI contributors if there are personal benefits to taking part in PPI |
|  | **Conducting PPI Activities** |
| **20** | There should be an induction session for everyone taking part in PPI |
| **21** | PPI activities should create opportunities for those involved to interact with one another |
| **22** | PPI activities should include identifying common areas of research interest between those involved |
| **23** | PPI activities should include identifying goals for preclinical/laboratory based spinal cord research |
| **24** | PPI activities should include opportunities for PPI contributors to share experiences of their condition |
| **25** | There should be at least two PPI meetings held every year for the duration of the project or study |
| **26** | PPI in preclinical spinal cord research should take place using virtual/hybrid platforms (i.e. Zoom/Teams) |
|  | **Evaluation and Dissemination** |
| **27** | PPI should be evaluated throughout the research project |
| **28** | Researchers should disseminate the impact PPI had upon their research |
| **29** | Researchers should include patient and public focused sections in preclinical research publications |
| **30** | PPI should involve developing information resources on preclinical research using language suited for non-scientific audiences |
|  | **Supporting PPI Contributors** |
| **31** | One member of the research team should be responsible for PPI within the project or study |
| **32** | Researchers should ensure funding is in place to support all aspects of PPI planned within the project |
| **33** | PPI contributors should be offered financial payment for their time taking part in PPI |
| **34** | PPI contributors should be offered funding to attend scientific conferences |
| **35** | Researchers should prioritise ease of access to in-person PPI activities |
| **36** | PPI contributors should be offered transport to/from in-person PPI activities |
| **37** | PPI contributors should be offered emotional support when taking part in PPI |
