# Supplementary material for: The Development of Principles for Patient and Public Involvement (PPI) in Preclinical Spinal Cord Research: A Modified Delphi Study
Source: Health Expect. 2024 Jul 4;27(4):e14130. doi: 10.1111/hex.14130 (PMC11222973; doi:10.1111/hex.14130)
Supplement: Supplementary file 4 — Supporting information. [file HEX-27-e14130-s002.docx]

**Appendix D:** Round 1 Summary Report

# Round one Delphi report

This report summarises the responses from the round one survey of the Delphi study. This survey was completed by a panel of 38 experts, 12 (31.6%) people affected by spinal cord injury, 15 (39.5%) researchers working in the area, and 11 (28.9%) doctors/surgeons working with patients living with spinal cord injury.

# Section 1: Recruiting PPI contributors

## Summary of round one responses


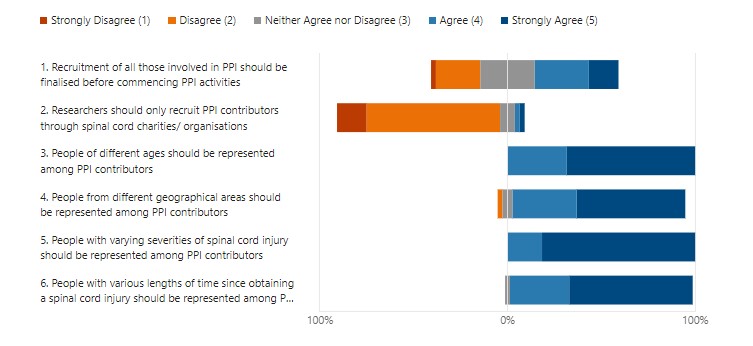


Statement 1: *“Recruitment of all those involved in PPI should be finalised before commencing PPI activities”* was scored as ‘agree’ or ‘strongly agree’ by 44.7% of panel members. Some panel members stated that finalising recruitment would ensure that PPI was in place from the beginning of the project or study, and would help with smooth and efficient running of PPI. However, many panel members indicated that people should be able to join at any time during the research project or study, so that no people are excluded from providing their input.

- Based on this, Statement 1 has been changed to: *“Recruitment of all those involved in PPI should ideally be finalised before commencing PPI activities, however this should be balanced against the risk of excluding people who would like to be involved.”*

Statement 2: *“Researchers should only recruit PPI contributors through spinal cord charities/ organisations”* was scored as ‘disagree’ or ‘strongly disagree’ by 86.9%. The main rationale provided for this was that recruiting PPI contributors solely through spinal cord charities or organisations may result in selection bias, as not all people with spinal cord injury are involved with such groups.

Statement 3: *“People of different ages should be represented among PPI contributors”* was scored as ‘agree’ or ‘strongly agree’ by 100%. The panel also suggested gender as a category to be represented amongst PPI contributors to ensure diverse perspectives.

- Based on this, Statement 3 has been changed to *“A range of demographics, e.g. in terms of age, gender, should be represented among PPI contributors.”*

Statement 4: *“People from different geographical areas should be represented among PPI contributors”* was scored as ‘agree’ or ‘strongly agree’ by 92.1%.

Statement 5: *“People with varying severities of spinal cord injury should be represented among PPI contributors”* was scored as ‘agree’ or ‘strongly agree’ by 100%. The main rationale for this was to represent a diversity of perspectives in PPI, and not just reflect a small subset of injuries.

Statement 6: *“People with various lengths of time since obtaining a spinal cord injury should be represented among PPI contributors”* was scored as ‘agree’ or ‘strongly agree’ by 97.4%.

The panel suggested that people affected by spinal cord injury should be informed of any physical demands of involvement in the project or study so they would not be dissuaded from being involved.

- Based on this, the following statement has been added – *New Statement A: “The recruitment process for PPI should explain what would be expected of people affected by spinal cord injury who agree to be involved, including any physical demands."*

**Section 2: PPI Training**

## Summary of round one responses


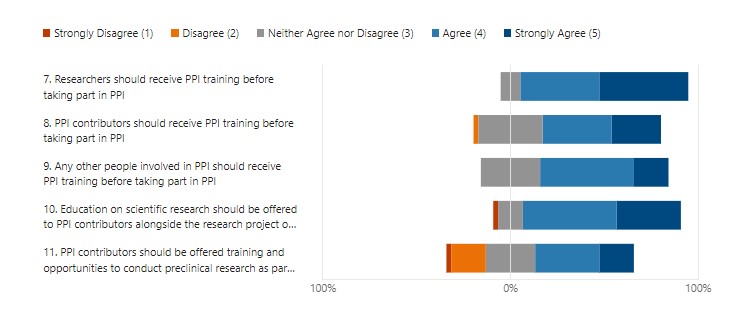


Statement 7: *“Researchers should receive PPI training before taking part in PPI”* was scored as ‘agree’ or ‘strongly agree by 89.5% of panel members. Some panel members felt that preclinical spinal cord researchers working primarily in laboratory settings may not be well-practiced in communicating with non-researchers as part of their work, and training would be beneficial for them.

Statement 8: *“PPI contributors should receive PPI training before taking part in PPI”* was scored as

‘agree’ or ‘strongly agree’ by 63.1%. Some panel members felt that training may bias PPI contributors, and it is their natural opinions that should be valued. Other panel members stated that it would depend on how PPI training was managed and delivered.

- Based on this, statement 8 has been changed to *“PPI contributors should receive PPI training to prepare and support them in contributing their own perspectives to the project or study.”*

Statement 9: *“Any other people involved in PPI should receive PPI training before taking part in PPI”* was scored ‘agree’ or ‘strongly agree’ by 68.4% of panel members. Some panel members stated that PPI training would help establish a common language between researchers, PPI contributors, and any other people involved.

Statement 10: *“Education on scientific research should be offered to PPI contributors alongside the research project or study”* was scored as ‘agree’ or ‘strongly agree’ by 84.2%. This was seen by some panel members as particularly important for preclinical research to reduce its abstract nature, and help PPI contributors become more familiar with the research project or study.

Statement 11: *“PPI contributors should be offered training and opportunities to conduct preclinical research as part of the research project or study”* was scored as ‘agree’ or ‘strongly agree’ by 52.6%. Some panel members believed that doing so was unlikely to be feasible for preclinical research taking place in laboratory settings, and may dissuade people from becoming involved in PPI.

- Based on this, Statement 11 has been changed to *“PPI contributors should be offered training and opportunities to carry out preclinical research as part of the project or study where practical and desired by the PPI contributors.”*

**Section 3: Agreeing on ways of working together**

Summary of round one responses


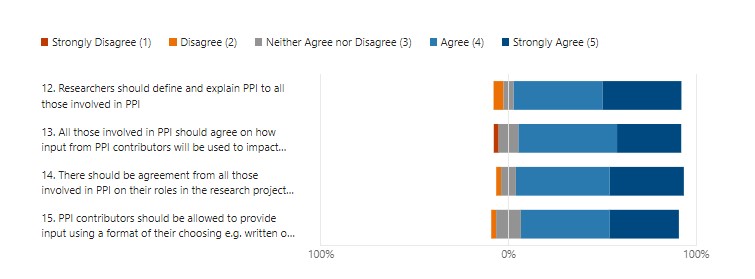


Statement 12: *“Researchers should define and explain PPI to all those involved in PPI”* was scored as ‘agree’ or ‘strongly agree’ by 89.5% of panel members.

Statement 13: *“All those involved in PPI should agree on how input from PPI contributors will be used to impact the research project or study”* was scored as ‘agree’ or ‘strongly agree’ by 86.8%.

Statement 14: *“There should be agreement from all those involved in PPI on their roles in the research project or study”* was scored as ‘agree’ or ‘strongly agree’ by 89.5%.

Statement 15: *“PPI contributors should be allowed to provide input using a format of their choosing e.g. written or verbal, virtual or in-person”* was scored as ‘agree’ or ‘strongly agree’ by 84.2%. Some panel members noted that this was particularly important for people living with spinal cord injury, as the severity of their injury may affect the way they can provide input.

# Section 4: Communication

## Summary of round one responses


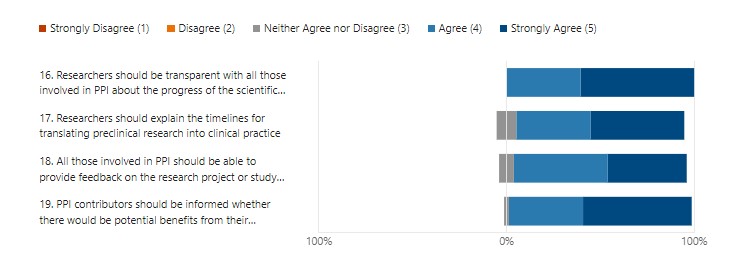


Statement 16: *“Researchers should be transparent with all those involved in PPI about the progress of the scientific research project or study”* was scored as ‘agree’ or ‘strongly agree’ by 100% of panel members.

Statement 17: “*Researchers should explain the timelines for translating preclinical research into clinical practice”* was scored as ‘agree’ or ‘strongly agree’ by 89.5%. Some panel members explained this was a key principle for PPI as timelines for translating research into clinical practice are long, risky, and undersold, which can be demoralising for PPI contributors.

Statement 18: *“All those involved in PPI should be able to provide feedback on the research project or study progress to researchers”* was scored as ‘agree’ or ‘strongly agree’ by 92.1%.

Statement 19: *“PPI contributors should be informed whether there would be potential benefits from their involvement in the research project or study, or the outputs it produces”* was scored as ‘agree’ or ‘strongly agree’ by 97.4%.

# Section 5: Activities for PPI in preclinical SCI research

## Summary of round one responses


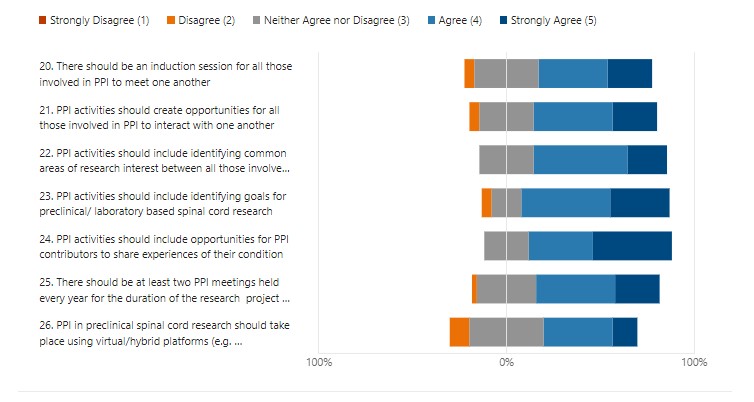


Statement 20: *“There should be an induction session for all those involved in PPI to meet one another”* was scored as ‘agree’ or ‘strongly agree’ by 60.5% of panel members. Some panel members felt that holding an induction session could foster a positive atmosphere and help people feel comfortable. It was suggested that induction sessions should be separate to the regular meetings.

• Based on this, Statement 20 has been changed to “*There should be an induction session for all those involved in PPI to meet one another, before commencing regular PPI meetings.”*

Statement 21: *“PPI activities should create opportunities for all those involved in PPI to interact with one another”* was scored as ‘agree’ or ‘strongly agree’ by 65.8%. Some panel members felt that interaction between researchers and PPI contributors would help establish a supportive network. Other panel members felt that PPI should focus on obtaining input on the research project or study, and that while creating opportunities for researchers and PPI contributors to interact was desirable, it may not be practical due to time constraints for both groups.

Statement 22: *“PPI activities should include identifying common areas of research interest between all those involved in PPI”* was scored as ‘agree’ or ‘strongly agree’ by 71.1%. Some panel members stated that this could be achieved by having the research teams present on their areas of interest.

Statement 23: *“PPI activities should include identifying goals for preclinical/ laboratory based spinal cord research”* was scored as ‘agree’ or ‘strongly agree’ by 79%. Some panel members reported that establishing shared goals would help develop the connections between researchers and PPI contributors. Other panel members stated that it may be difficult to establish shared goals for preclinical research, and that such goals would typically be established before the PPI takes place.

Statement 24: *“PPI activities should include opportunities for PPI contributors to share experiences of their condition”* was scored as ‘agree’ or ‘strongly agree’ by 76.3%. Some panel members expressed that they would like to understand the backgrounds of other people involved in the project or study. Other panel members felt that social interaction was not a primary focus for PPI.

Statement 25: *“There should be at least two PPI meetings held every year for the duration of the research project or study”* was scored as ‘agree’ or ‘strongly agree’ by 65.8%. Some panel members indicated that scheduling too many meetings may be burdensome, and that 1-2 meetings per year would be sufficient. Other panel members believed that more meetings would improve PPI contributors understanding of the potential outcomes of the research project or study.

Statement 26: *“PPI in preclinical spinal cord research should take place using virtual/hybrid platforms (e.g. Zoom/Teams)”* was scored as ‘agree’ or ‘strongly agree’ by 50%. Some panel members reported that in-person meetings would have improved engagement over virtual/hybrid platforms. Other panel members highlighted that using online platforms would make it easier for people with mobility issues to be involved. Hybrid options were also considered, though some panel members stated that hybrid sessions made it harder for the online attendees to feel equal to those attending in-person.

**Section 6: Evaluating and disseminating PPI**

## Summary of round one responses


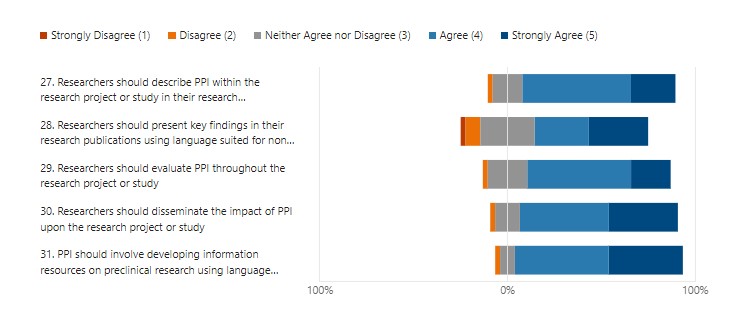


Statement 27: *“Researchers should describe PPI within the research project or study in their research publications”* was scored as ‘agree’ or ‘strongly agree’ by 81.6% of panel members. Many panel members felt this would help normalise PPI and give it more visibility. Other panel members felt that journals which use well established publication frameworks and guidelines may make describing PPI difficult in a study.

Statement 28: *“Researchers should present key findings in their research publications using language suited for non-scientific audiences”* was scored as ‘agree’ or ‘strongly agree’ by 60.5%. This principle was contentious amongst panel members, with some stating all published findings should use language familiar to non-scientific audiences, while other panel members reported that oversimplifying research findings may diminish their meaning.

Some panel members suggested that key findings published in journals should use language tailored for researchers, and that researchers should then have separate routes for dissemination e.g. charities or social media, and present their findings using more general language there.

• Based on this, the following statement has been added *New Statement B: “Key research findings published in journals should also be disseminated using routes and language suited for non-scientific audiences”*

Statement 29: *“Researchers should evaluate PPI throughout the research project or study”* was scored as ‘agree’ or ‘strongly agree’ by 76.4%.

Statement 30: *“Researchers should disseminate the impact of PPI upon the research project or study”* was scored as ‘agree’ or ‘strongly agree’ by 84.2%.

Statement 31: *“PPI should involve developing information resources on preclinical research using language suited for non-scientific audiences”* was scored as ‘agree’ or ‘strongly agree’ by 89.5%. Some panel members stated this principle would help bridge the gap between patients and the research conducted on their behalf.

**Section 7: Supporting PPI contributors**

## Summary of round one responses


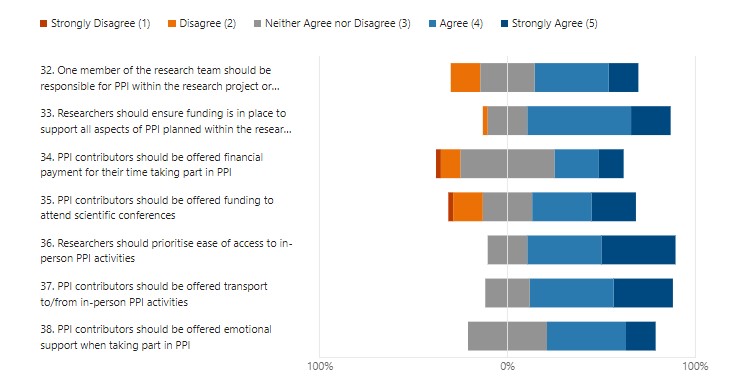


Statement 32: *“One member of the research team should be responsible for PPI within the research project or study”* was scored as ‘agree’ or ‘strongly agree’ by 55.5% of panel members. Some panel members explained that having one researcher in charge would ensure time and resources were dedicated to PPI, and create a focal point for PPI contributors. Other panel members stated that multiple researchers should be responsible for PPI, as designating one person to be in charge would be overly-prescriptive.

Statement 33: *“Researchers should ensure funding is in place to support all aspects of PPI planned within the research project or study”* was scored 76.4% ‘agree’ or ‘strongly agree.’ Panel members stated that covering costs would be essential, and to not do so would be a disservice to PPI contributors.

Statement 34: *“PPI contributors should be offered financial payment for their time taking part in PPI”* was scored 36.9% ‘agree’ or ‘strongly agree.’ Panel members were divided on this statement. Some thought that paying PPI contributors may provide the wrong incentive to be involved, bias PPI contributors and that it would be difficult to source funding for payments. Others members believed that paying a fee to PPI contributors for their time would improve the participation of people in PPI.

Statement 35: *“PPI contributors should be offered funding to attend scientific conferences”* received a score of 55.3% ‘agree’ or ‘strongly agree.’ Some panel members reported that PPI contributors should be offered funding to attend conferences because they contribute to producing research.

Statement 36: *“Researchers should prioritise ease of access to in-person PPI activities”* was scored 79% ‘agree’ or ‘strongly agree.’

Statement 37: *“PPI contributors should be offered transport to/from in-person PPI activities”* received a score of 76.3% ‘agree’ or ‘strongly agree.’ Panel members supported this principle on the basis that that there should not be a cost to being a PPI contributor. This was seen as particularly relevant for people affected by spinal cord injury who may be required to arrange specialised transport and accommodation.

Statement 38: *“PPI contributors should be offered emotional support when taking part in PPI”* scored 57.9% ‘agree’ or ‘strongly agree.’ Panel members reported that emotional support would be important due to the personal nature of a spinal cord injury. Other panel members felt emotional support was desirable, but would only impact a small number of PPI contributors.
